# EST sequencing and gene expression profiling of defence-related genes from *Persea americana *infected with *Phytophthora cinnamomi*

**DOI:** 10.1186/1471-2229-11-167

**Published:** 2011-11-23

**Authors:** Waheed Mahomed, Noëlani van den Berg

**Affiliations:** 1 Forestry and Agricultural Biotechnology Institute (FABI), University of Pretoria, Pretoria, 0002, South Africa; 2 Department of Genetics, University of Pretoria, Pretoria, 0002, South Africa

## Abstract

**Background:**

Avocado (*Persea americana*) belongs to the Lauraceae family and is an important commercial fruit crop in over 50 countries. The most serious pathogen affecting avocado production is *Phytophthora cinnamomi *which causes Phytophthora root rot (PRR). Root pathogens such as *P. cinnamomi *and their interactions with hosts are poorly understood and despite the importance of both the avocado crop and the effect *Phytophthora *has on its cultivation, there is a lack of molecular knowledge underpinning our understanding of defence strategies against the pathogen. In order to initiate a better understanding of host-specific defence we have generated EST data using 454 pyrosequencing and profiled nine defence-related genes from Pc-infected avocado roots.

**Results:**

2.0 Mb of data was generated consisting of ~10,000 reads on a single lane of the GS FLX platform. Using the Newbler assembler 371 contigs were assembled, of which 367 are novel for *Persea americana*. Genes were classified according to Gene Ontology terms. In addition to identifying root-specific ESTs we were also able to identify and quantify the expression of nine defence-related genes that were differentially regulated in response to *P. cinnamomi*. Genes such as *metallothionein, thaumatin *and the *pathogenesis related PsemI, mlo *and *profilin *were found to be differentially regulated.

**Conclusions:**

This is the first study in elucidating the avocado root transcriptome as well as identifying defence responses of avocado roots to the root pathogen *P. cinnamomi*. Our data is currently the only EST data that has been generated for avocado rootstocks, and the ESTs identified in this study have already been useful in identifying defence-related genes as well as providing gene information for other studies looking at processes such as ROS regulation as well as hypoxia in avocado roots. Our EST data will aid in the elucidation of the avocado transcriptome and identification of markers for improved rootstock breeding and screening. The characterization of the avocado transcriptome will furthermore form a basis for functional genomics of basal angiosperms.

## Background

Avocado (*Persea americana *Mill.) is an important agricultural crop in over 50 countries worldwide and is native to Mexico and Central America [1]. It belongs to the genus-*Persea*, subgenus-*Persea*, family-*Lauraceae *and falls under the clade of magnoliids that are sister to eudicot and monodicot clades. *P. americana *is a diploid angiosperm consisting of 24 chromosomes with approximately 8.83 × 10^8 ^(883 Mb) base pairs (bp). To date, the avocado genome is not yet available and only a limited number (16558) of expressed sequence tags (ESTs) generated from only fruit and flowers have been sequenced, annotated and released on the NCBI database.

Phytophthora root rot (PRR), caused by *Phytophthora cinnamomi *Rands, is considered the most destructive pathogen-induced disease to the avocado industry [2-4] with production relying heavily on the use of phosphite trunk injections and tolerant rootstocks such as Dusa^® ^[4,5] supported by planting in high organic matter soils and mulching to promote antagonistic microbial growth against *P. cinnamomi*. Metalaxyl has also showed promising results when used in conjunction with the tolerant rootstock Duke 7 in California in the 1980s [6]. However in South Africa it was reported that after successful application for a period of two years, metalaxyl application became inefficient in controlling the disease [7]. Recently *P. cinnamomi *has shown a decrease in sensitivity to phosphite treatments after prolonged usage [8]. The authors demonstrated that *P. cinnamomi *isolates exposed to long periods of phosphite treatment in south west Australia showed reduced sensitivity to the fungicide when evaluated on avocado, lupins and eucalyptus. The population of *P. cinnamomi *isolated from phosphite treated sites colonized phosphite treated plant material easier than isolates not previously exposed to the fungicide. This decreased sensitivity to phosphite could indicate the onset of resistance to the fungicide.

As early as 1926 avocado researchers identified that the success of the avocado industry lay in rootstock improvement [9]. The world's largest rootstock germplasm is maintained in California since 1957, with the hope of identifying more tolerant rootstocks for cultivation [6,10]. To date a small number of rootstocks have been identified with partial resistance to *P. cinnamomi *such as Thomas, Martin Grande, Barr Duke, Duke 7 and D9 [11]. In South Africa, the devastation caused by *P. cinnamomi *in the 1970s prompted the importation of clonal rootstocks and the development of a large scale selection program in the 1980s. For many years Duke 7 remained the industry standard in South Africa, until 2002 with the release of the Dusa^® ^rootstock by Westfalia Technological Services. Dusa^® ^gave avocado farmers a better alternative to Duke 7 that showed improved tolerance to *P. cinnamomi *as well as good fruit yields. The avocado breeding program at Westfalia is a continuing process and uses previously identified tolerant rootstocks as parents to undergo open pollination. Recently, field trials were conducted on a selection of rootstocks in Queensland, Australia with some selections such as 'SHSR-02', 'SHSR-04', un-grafted 'Hass' and Dusa^® ^demonstrating their tolerance to PRR [12].

Despite the importance of avocado and a 60 year attempt to unravel the host pathogen interaction, our knowledge is based on; the analysis of root exudates[13], chemical analysis of roots [12], the application of chemicals to aid in suppression of the pathogen [14], and biochemical studies [15]. Histological studies on roots infected with *P. cinnamomi *have aimed to try and understand the plant pathogen interaction [16]. It was observed that necrophylactic periderm and periclinal cell wall division occurred, which limited the pathogens progress but did not affect the viability of the pathogen or reduce its ability to infect the host plant. *P. cinnamomi *infect the plants roots via motile zoospores present in the soil. The attraction of zoospores was investigated by Botha and Kotze in 1989 and it was found to be influenced by the composition of 14 amino acids in avocado root exudates [13]. Sánchez-Pérez and colleagues tested crude root exudates for *P. cinnamomi *mycelial inhibition and subsequently the compound known as stigmastan-3, 5-diene was identified as the inhibitory compound [12]. García-Pineda *et al*. (2010) investigated reactive oxygen species (ROS) formation and the role of nitric oxide (NO) against *P. cinnamomi *[15]. The authors observed an increase in ROS and NO levels and deduced that the increase in ROS observed may assist in weakening host tissue early in infection with the sharp increase in NO possibly resulting in salicylic acid (SA) accumulation. This accumulation could cause an SA mediated H_2_O_2 _burst by the suppression of H_2_O_2 _degradation. The authors hypothesize that (cytosolic tobacco catalase) CAT is bound by SA, which inhibits CATs H_2_O_2 _degrading activity. The effect of externally applied SA on root colonisation was also investigated and indicated that decreased root colonisation was associated with SA application. SA has been implicated in regulating cell death, inducing resistance responses and activating various defence genes such as pathogenesis-related (PR) genes [17] but the mode of action has not been elucidated. The production of NO and ROS have previously been demonstrated to activate cell death. These early attempts on investigating the interaction between avocado and *P. cinnamomi *have illustrated the complexity of the defence response, highlighting the need for the molecular elucidation of defence genes.

Molecular research on avocado has comprised of genetic relationship studies and the molecular characterization of the fruit and flowers. There has been some gene characterization of avocado fruit ripening genes [18-22]. The greater part of molecular detail exists due to a continuous effort in marker development to assist in either elucidating genetic relationships amongst scions [23-28], or scion improvement [29-32]. There is currently a preliminary genetic map available based on microsatellites, random amplified polymorphic DNA (RAPD) markers and DNA fingerprint (DFP) markers [33]. The most recent molecular development in the fight against PRR was the identification of 70 microsatellite markers that were developed from over 8000 ESTs in the hope of aiding in marker assisted breeding against PRR. The ESTs were however from a floral gene database generated for comparative genomics research of basal angiosperms. Their efficacy has yet to be tested for use in identifying tolerant rootstocks, but it is known that they amplify across all avocado varieties and can be used for investigation of genetic relations [34,35]. The University of California Riverside (UCR) has recently employed 61 polymorphic AFLP markers to characterise PRR tolerance in 83 rootstocks from various locations including South Africa and Israel with the majority of rootstocks from the UCR collection [36]. The study concluded that resistance mechanisms vary between tolerant cultivars and no trend was observed in the cluster analysis.

The avocado/*P. cinnamomi *interaction has not previously been elucidated on a molecular level. Current knowledge is based on research of the non-host plant, *Arabidopsis*. A study conducted on *Arabidopsis *infected with *P. cinnamomi *revealed that ROS induction, HR activation, lignin synthesis and callose production was initiated upon infection. The non-host showed activation of the ethylene and jasmonic acid pathways and only a minor involvement of the SA pathway [37] in contrast to the study conducted by García-Pineda *et al *(2010) on avocado which indicated that SA is a major inhibitor of pathogen colonisation. Macroscopic changes such as callose production have also been observed during *P. cinnamomi *infection in maize [38] and although model plants like *Arabidopsis *provide an insight to defence responses there are differences between non-host and host-specific defence responses. In order to fully understand tolerance in avocado it is important to conduct molecular level studies on the host specific interaction between *P. americana *and *P. cinnamomi*.

Since there is no genome data available for avocado, the identification and characterisation of genes is difficult. EST generation supplements the lack of genome data by providing transcript specific information and excluding non-coding regions of the genome. High-throughput sequencing is well suited for large scale EST discovery, providing a tool for gene discovery in non-model crops to evaluate the changes in gene expression to abiotic or biotic stresses [39-41]. The cost of pyrosequencing is also lower than conventional EST sequencing, small transcripts are not lost, and the time in sequence generation from tissue isolation is greatly shortened [39]. More specifically, it is advantageous for commercial crops that lack substantial molecular databases and will aid in their unconventional improvement [40]. Avocado is one such commercial crop that is in need of development of molecular tools for the improvement of the crop. Avocados' importance as an agricultural crop has justified molecular investigation and the application of modern molecular tools for its improvement [26,27,31,33,42,43]. The application of high-throughput sequencing to avocado is the next step in improving breeding of this economically important crop.

In this study ESTs of a tolerant avocado rootstock infected with *Phytophthora cinnamomi *were generated. The 454 GS-FLX platform was used to generate sequence data for several time points including the uninfected, 6, 12, 24, 48 and 72 hours after infection, as well as to identify transcripts that were associated with the defence response. We identified 371 transcripts from avocado and studied the gene expression of a selection of these ESTs, thereby providing the first molecular data for the avocado/*P. cinnamomi *interaction.

## Results

### 454 pyrosequencing and assembly

**Figure 1:**
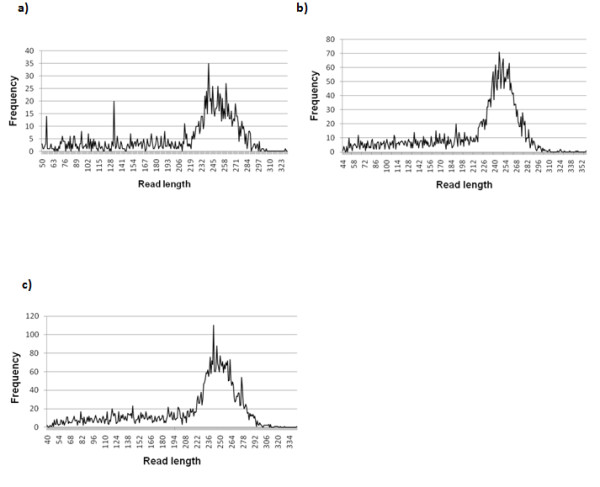
**Read length distributions of uninfected, library 1 and library 2 infection Dusa^® ^cDNA**. Pyrosequencing was performed on the 454 GS-FLX platform **(a**) Uninfected library contains reads with the highest frequency at around 245 bp. **(b) **Library 1 reads have the highest frequency at around 252 bp. **(c) **Library 2 reads have the highest frequency at around 240 bp.

Three cDNA libraries-uninfected (0 hr), library 1 (6 & 12 hr) and library 2 (24, 48 & 72 hr) were sequenced on a single lane on the GS FLX platform and generated a total of 2 Mb of data (after trimming and quality control) consisting of 9953 reads and resulted in the assembly of 371 contigs (Table 1). These contigs comprised of 1407 reads from the uninfected library, 3584 reads from infection library 1 and 4962 reads from infection library 2. The average read lengths for the libraries were 216.4 bp for uninfected, 217.5 bp for library 1 and 215.9 bp for the library 2 (Figure 1). The pyrosequencing run was efficient based on the maximal amount of data obtainable being 2.5 Mb with a maximal read length of 250 bp.

**Table 1:** Excerpts of newblermetric reports from the uninfected, library 1 and library 2 libraries of *Phytophthora cinnamomi *infected avocado roots.

	Uninfected response	Library 1	Library 2
Total Reads	1407	3582	4961
Total Bases	288885	737254	1017064
Number of Contigs	43	139	189

The total number of reads and contigs are shown to illustrate the efficiency of the pyrosequencing run.

### EST identification and classifications

After analysis using the dCAS software, 367 novel ESTs were identified for *P. americana*. The program used BLASTX amino acid comparisons to screen for homology of the contigs against the NCBI non-redundant (NR) database. A large proportion of the sequences generated showed homology to hypothetical proteins and 45 of the 371 contigs had no similarity to previously annotated sequences (Table 2). Of the 371 contigs identified, only two sequences showed homology to previously identified fructose-bisphosphate aldolase and metallothionein type-II proteins from avocado, with the remaining 369 not having any avocado sequence homolog in the NR database. Manual BLAST annotation did not influence transcript identification.

**Table 2:** Contig classification for cDNA libraries of *Phytophthora cinnamomi *infected avocado roots.

	Uninfected	Library 1	Library 2	Total
**Unidentified**	5	16	24	45
**Hypothetical protein**	23	54	75	152
**Genes identified**	15	69	89	173

Contigs were classed as unidentified, identified or hypothetically identified if the sequence homology search revealed that there was no similarity, significant similarity or inferred structural function respectively. The total number of genes identified was 173 with only 45 of the total 370 contigs generating no identification using the non-redundant (NR) database on the NCBI.

**Figure 2:**
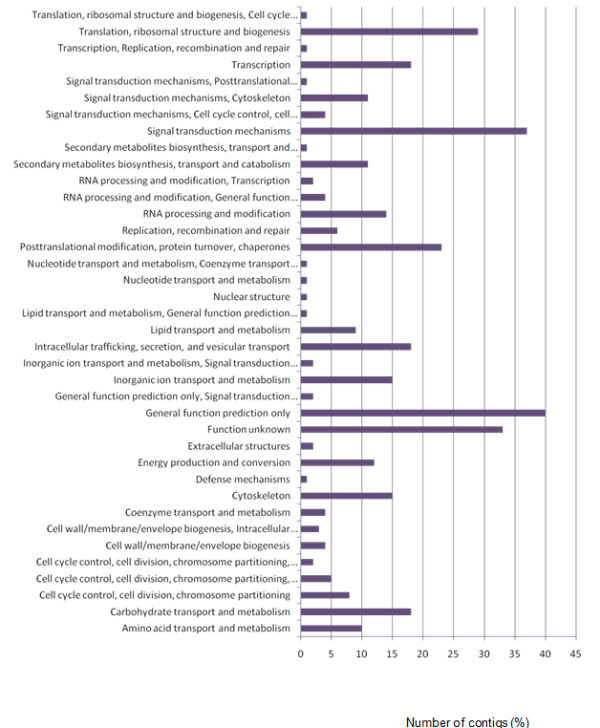
**KOG (euKaryotic Orthologous Groups) classifications of avocado transcripts identified in three cDNA libraries**. The contigs generated from the 454 data were compared against the KOG database to assign functional classifications.

Contigs were grouped into functional classes according to the GO (Gene Ontology) and KOG (Eukaryotic Orthologous Groups) databases. Nine percent of contigs were grouped into the unknown functional class in the KOG database while 44.5% of contigs from the GO classification were represented by unknown functions (Figure 2 & Table 3). The categories of post-translational modification; translation, ribosomal structure and biogenesis; signal transduction mechanisms and general function prediction contained a combined total of 34.8% of all contigs. According to the GO database the functional classifications of cell wall related; protein binding; stress response; ribosomal structural constituent; cytoplasmic biological processes; cellular component and other categories comprised 40.8% of all contigs with 3% (12/371) of the contigs linking directly with stress responses. Over 20 putative defence related genes were identified ranging from general defence-related genes (*metallothioneins, thaumatin *and *universal stress *genes) to more specific oomycete defence-related genes (*pathogenesis related protein *PR10 and the *oxysterol-binding *gene) (Tables 4&5).

**Table 3:** Contigs of avocado transcripts grouped into functional classes according to GO database.

Gene ontology	Number of contigs
Unknown	165
Other	48
ATP binding	7
Biological process - cytoplasm	20
Cellular component	39
Response to stress	12
RNA binding	5
Cell wall related	10
Structural constituent of ribosome - translation - ribosome	12
Transcription factor activity - regulation of transcription	7
Transferase activity/cell wall biogenesis	4
Translation elongation factor activity - translation factor activity, nucleic acid binding	3
Transporter activity - transport	3
Water channel activity - transport - membrane	4
Protein binding	10
Mitochondrion	9
Kinase activity	4
Protein folding - cellular component	3
Membrane	3
Lipid binding - lipid transport	2

A large proportion of contigs (44.5%) fell into the category of unknown classification while contigs that link directly with stress responses constituted 3% of the total number of contigs.

**Table 4:** List of putative stress related genes isolated from *Phytohpthora cinnamomi *infected avocado roots.

Contig	Putative identity	E Value	Species
**library 1 library**			
contig0020	*leucine-rich repeat resistance protein-like protein*	8e^-15^	*Gossypium hirsutum*
contig0070	*4-coumarate-CoA ligase-like protein*	5e^-26^	*Arabidopsis thaliana*
contig0088	*seven transmembrane protein Mlo*	1e^-28^	*Zea mays*
contig0106	*pathogenesis-related protein PsemI*	5e^-14^	*Pseudotsuga menziesii*
contig0109	*drought-induced protein*	8e^-13^	*Retama raetam*
contig0076	*metallothionein-like protein type 2*	6e^-41^	*Persea americana*
**library 2 library**			
contig00007	*translationally controlled tumour protein like protein*	2e^-07^	*Nicotiana tabacum*
contig00011	*thaumatin*	2e^-20^	*Vitis riparia*
contig00043	*cinnamate-4-hydroxylase*	3e^-16^	*Gossypium arboreum*
contig00064	*metallothionein-like protein type 2*	7e^-41^	*Persea americana*
contig00065	*AP2 domain containing protein*	2e^-41^	*Prunus armeniaca*
contig00073	*oxysterol-binding protein*	3e^-28^	*Solanum tuberosum*
	*thaumatin-like protein, putative*	3e^-29^	*Arabidopsis thaliana*
contig00095	*AP2 domain containing protein*	7e^-27^	*Prunus armeniaca*
contig00108	*profilin-like protein*	5e^-17^	*Cinnamomum camphora*
contig00163	*Translationally-controlled tumour protein homolog (TCTP) translationally controlled tumour protein*	1e^-29^	*Hevea brasiliensis*
contig00169	*cysteine proteinase*	8e^-39^	*Elaeis guineensis*
contig00175	*putative universal stress protein*	2e^-40^	*Cicer arietinum*
contig00187	*cytochrome P450 like TBP*	2e^-60^	*Nicotiana tabacum*
contig00054	*metallothionein-like protein class II*	4e^-39^	*Nelumbo nucifera*
contig00057	*dormancy/auxin associated family protein*	6e^-15^	*Arabidopsis thaliana*
contig00081	*putative aquaporin PIP2-1*	5e^-76^	*Vitis berlandieri × Vitis rupestris*

Six genes were isolated from library 1 cDNA while 15 genes were identified from the library 2 cDNA. These transcripts play a role in either biotic or abiotic stress responses showing the species to which the sequence showed homology.

**Table 5:** Defence-related genes isolated from *Phytophthora cinnamomi *infected avocado roots.

Gene	E-value		Response against
*thaumatin*	2e^-20^	*Vitis riparia*	*Armellaria mellea *[65]
*metallothionein-like protein type 2*	7e^-41^	*Persea americana*	*Agrobacterium rhizogenes *[53]
*pathogenesis-related protein P sem I*	5e^-14^	*Pseudotsuga menziesii*	*Phellinus weirii *[49]
*putative universal stress protein*	2e^-40^	*Cicer arietinum*	-
*profilin-like protein*	5e^-17^	*Cinnamomum camphora*	*Phytophthora infestans *[60]
*oxysterol-binding protein*	3e^-28^	*Solanum tuberosum*	*Phytophthora *spp [66]
*LRR resistance protein-like protein*	8e^-15^	*Gossypium hirsutum*	*Phytophthora infestans *[64]
*seven transmembrane protein Mlo*	1e^-28^	*Zea mays*	*Blumeria graminis *f. sp. *hordei *[62]

Certain transcripts could be related directly to oomycete infection in other plants. Strong E-values indicate confidence in the identification of these defence-related genes.

### Species similarity between avocado and other plants

**Figure 3:**
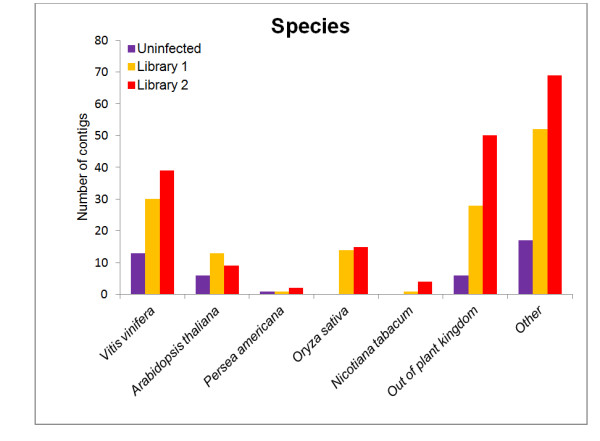
**Number of contigs grouped according to sequence homology between avocado and other plant species**. The sequence similarities were analysed to establish which species was most represented by the 454 data. There is an observable lack of avocado sequence data available on public databases.

We observed significant sequence homology between *Vitis vinifera *(grape) and avocado when the species origin of the sequence similarity was investigated. The top three represented species according to amino acid homology on the NCBI were *V. vinifera, Arabidopsis thaliana *and *Oryza sativa*, with *V. vinifera *having the majority of the hits in all three libraries. Twenty two percent of sequences showed homology to *V. vinifera *sequences with 7.5% belonging to *A. thaliana *and 7.8% of sequences to *O. sativa*. Homology to *P. americana *was found in only 1% of sequences (4/371) (Figure 3). Only two genes were represented by the 1% in which the metallothionein transcript featured three times and fructose-bisphosphate aldolase featured once. Grape vine featured among the top ten homologous hits of every contig that was annotated. Thirty seven percent of the annotated contigs were represented by various plant species such as *Prunus armeniaca*, *Solanum tuberosum*, *Hevea brasiliensis *with the variety of plant species not biased to any particular family or order. The majority of the species similarities relate to a large variety of plants that have been collectively categorised as other.

### Quantitative gene expression analysis

Expression analyses of nine genes were conducted at 0, 3, 6, 12, 24 and 48 hours post infection (hpi) to validate if the pyrosequencing data reflected their gene expression. This was normalized against 18S and actin reference genes to give the relative gene expression. The expression data was then compared against the pyrosequencing data which revealed that six of the nine genes showed similarities between the two methods, showing the highest expression at a time point belonging to the library from which the transcript emanated (Table 6).

**Table 6:** Similarities between pyrosequencing data and gene expression profiles of defence-related genes

Sequence ID	GenBank accession number	cDNA Library	Max qRT-PCR expression	Similarities between 454 and qRT-PCR
*Thaumatin *	JO840464	Library 2	48 hpi	yes
*LRR resistance PLP *	JO840460	Library 1	12 hpi	yes
*Metallothionein like protein *	JO840461	Uninfected, Library 1	12 hpi	yes
*Thaumatin-like protein *	JO840465	Library 2	12 hpi	yes
*Seven transmembrane protein MI0 *	JO840462	Library 1	12 hpi	yes
*Pathogenesis-related protein PsemI *	JO840463	Library 1	24 hpi	no
*Proflin-like protein *	JO840466	Library 2	12 hpi	no
*Putative universal stress protein *	JO840467	Library 2	12 hpi	no
*Cytochrome P450 like TBP *	JO840468	Library 1 and Library 2	3/12 hpi	yes

All the genes chosen for expression profiling from the tolerant avocado rootstock infected with *Phytophthora cinnamomi *showed the highest expression from a time point related to the cDNA library of their identification except the pathogenesis-related protein, profilin-like protein and the universal stress protein.

**Figure 4:**
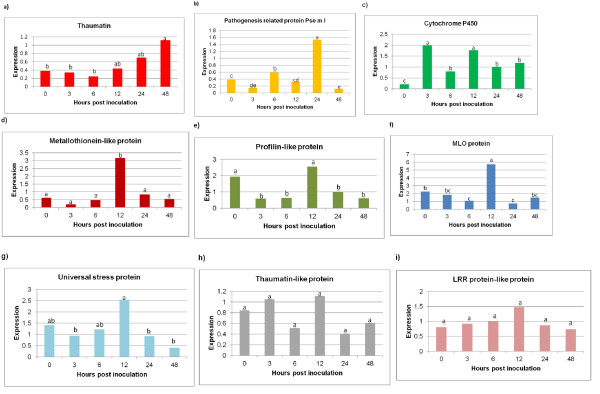
**Gene expression of Dusa^® ^-a tolerant avocado rootstock, infected with *Phytophthora cinnamomi***. Expression analysis was conducted at 0, 3, 6, 12, 24 & 48 hpi (hours post infection) with 0 hr being the uninfected control. The data was normalized using two reference genes-actin and 18S. Expression analysis was performed in triplicate on three biological replicates **(a) ***Thaumatin*. **(b) **Pathogenesis-related protein *psemI *(PR10). **(c) ***Cytochrome P450*. **(d**) *Metallothionein-like *gene. **(e) ***Profilin-like *gene. **(f) ***MLO *transmembrane gene. **(g) **The universal stress protein. **(h) **The *thaumatin-like *gene. **(i) **The LRR resistance protein-like protein.

Thaumatin expression was significantly greater at 48 hpi (1.1) as oppose to the uninfected (0.4), as well as the 3 & 6 hpi (Figure 4a). The expression pattern indicated that thaumatin was only regulated in response to *P. cinnamomi *by 12 hpi and increased by nearly threefold over a 36 hour period. Thaumatin levels were significantly higher in the later infection time points when compared to the earlier time points-thus correlating with the pyrosequencing data.

The pathogenesis-related (PR-10) *psemI *gene showed significant increases in expression at 6 & 24 hpi. At 24 hpi *psemI *reached the highest expression level of 1.5 when compared to all time points (Figure 4b). By 48 hpi *psemI *expression had decreased significantly to 0.1, reaching levels comparable to 3 hpi.

Cytochrome P450-like TBP (TATA box binding protein) showed a significant early response at 3 hr after infection with *P. cinnamomi *reacting with an increase of ten-fold. At 6 hpi the gene was significantly down-regulated followed by a substantial increase at 12 hpi- a similar level found at 3 hpi. The gene was then significantly down-regulated to 1.0 at 24 hpi and remained unchanged at 48 hpi (Figure 4c). Cytochrome P450-like TBP levels were constantly up- and down-regulated showing significant variation over time points. The data was consistent with the pyrosequencing data for this transcript.

The gene encoding for a metallothionein-like protein was constitutively expressed at 0.6 and showed no significant changes in expression over the first 6 hpi. This was however followed by a significant increase in the expression at 12 hpi when compared to all other time points reaching levels of 3.2, the expression then decreased to 0.5 at 24 hpi and remained unchanged at 48 hpi (Figure 4d). The data showed similarity to the pyrosequencing data for this transcript.

The *profilin-like *gene was expressed constitutively at 1.9 prior to infection. Three hours after infection the transcript was significantly down-regulated to 0.6 (a 3 fold decrease) and remained unchanged at 6 hpi. There was a significant up-regulation from 6 hpi to12 hpi with expression peaking at 2.6, followed by a significant decrease to 0.9 at 24 hpi and remained unchanged at 48 hpi as opposed to 12 hpi (Figure 4e).

The MLO transmembrane protein encoding gene was constitutively expressed at 2.2 followed by a significant reduction at 6 hpi compared to the uninfected time point. At 12 hpi the tolerant rootstock responded with a significant increase to 5.7. Expression at 24 hpi was significantly down-regulated when compared to 12 hpi and reached a level of 1.5 at 48 hpi (Figure 4f). The *mlo expression *data was in agreement with the pyrosequencing data for this transcript.

The universal stress protein showed the maximum expression at 12 hpi but did not show a significant increase when compared to 0 and 6 hpi. An overall increase in expression was viewed until 12 hpi, followed by a significant down-regulation to 0.4 at 48 hpi compared to 12 hpi (Figure 4g).

Two genes encoding respectively for the thaumatin-like protein and the leucine rich repeat (LRR) resistance protein-like protein were constitutively expressed at 0.8. Both genes showed no statistically significant change in regulation over the 48 hour time course, however similar to the majority of genes in this experiment, both genes showed the highest increase in expression at 12 hpi. The highest level of up-regulation achieved was 1.1 and 1.47 respectively (Figure 4h &4i).

## Discussion

We have sequenced the first set of avocado root transcriptomic data for the avocado-*P. cinnamomi *interaction. A single lane of pyrosequencing on the GS FLX platform generated 2.0 Mb (of a potential 2.5 Mb) of data, consisting of 9953 reads that assembled into 371 contigs of which 367 ESTs are novel for *P. americana *and have not previously been identified. In addition, we were also able to identify and quantify the expression of nine defence-related genes that were regulated in response to *P. cinnamomi*. The primary objective of this study was to generate EST data of a tolerant avocado rootstock infected with the root pathogen *P. cinnamomi*. This data identified the genes involved in cellular processes and defence mechanisms thereby providing the first platform for studying molecular mechanisms underlying tolerance in the roots of one of the important agricultural hosts of *P. cinnamomi*.

The 371 contigs were grouped into 38 and 21 functional classes based on the KOG and GO databases respectively using the dCAS program. As expected, the majority of sequences had unknown functions. Due to the high sensitivity of sequencing, transcriptome studies identify many transcripts that have not yet been characterised and many that have unknown functions even when annotated using a database such as Gene ontology [44]. The lack of EST and genome sequence data for avocado in general, specifically rootstocks, also accounts for the high frequency of unknown functions. The top ten functional groupings according to the GO classification revealed that 44.5% of assembled contigs were represented by unknown functions followed by the functional groups of 'other', 'cellular components', 'biological processes', 'stress responses', 'ribosome structure', 'cell wall related', 'protein binding', 'mitochondrion and 'ATP-binding'. According to the KOG database much of sequence data matched categories of 'general function prediction', which means that these transcripts show homology to transcripts that are poorly characterised according to the NCBI. The KOG database revealed that the top ten classes that the contigs grouped into were firstly; 'general function prediction' followed by 'signal transduction', 'unknown function', 'translation and ribosomal structure', 'chaperones', 'carbohydrate metabolism', 'intracellular trafficking', 'transcription', 'cytoskeleton' and 'inorganic ion transport and metabolism'. Furthermore, the presence of unidentified reads in this study is not unique to avocado, other studies have also produced sequence that did not align to any sequence present in NCBI datasets [39].

We investigated the sequence homology between the avocado sequence data and the plant species that our data showed homology to. Only 1% (4/371) of the sequenced contigs showed homology to *P. americana*, while 20-30% of the contigs generated showed similarity to grapevine (*V. vinifera*). The lack of similarities to any avocado sequence data observed in our study emphasizes the lack of genetic data on the NCBI. The knowledge we have gained by sequencing avocado rootstock ESTs may provide some insight into other magnoliids or phylogenetically related plants. The sequence and expression data generated in this study can form a basis for functional genomics of basal angiosperms - a group which has no other model [27].

As expected we isolated a number of ESTs with homology to genes previously associated with defence responses in plants against pathogens and in some cases, against oomycetes. Interesting defence ESTs included thaumatin, metallothionein, a PR10 pathogenesis-related protein, a mlo transmembrane protein and profilin. Nine genes were quantified with qRT-PCR to elucidate the early gene response of a tolerant avocado rootstock infected with *P. cinnamomi *as well as to validate the pyrosequencing data.

Thaumatin, a PR5 protein associated with the SA pathway [45,46], was significantly upregulated at 48 h in response to *P. cinnamomi *infection. The gene showed no changes in regulation during the first 6 hours after inoculation with a mycelial suspension. At 12 and 24 hpi expression showed an insignificant but steady increase in response to the infection. PR5 is induced by biotic stress and further linked to increased pathogen resistance [47]. García-Pineda *et al *(2010) showed decreased root colonization in the *Arabidopsis-P. cinnamomi *system linked to SA. The significant up-regulation of thaumatin in the *P. cinammomi *tolerant avocado rootstock indicates the importance of the SA pathway in the early inhibition of the hemibiotroph *P. cinnamomi*. Hemibiotrophs have an initial biotrophic phase prior to becoming necrotrophs and PR5 gene activity in the SA-dependant pathway has been previosuly shown to be effective against biotrophs [48].

*PsemI *was highly expressed at 24 hpi in tolerant avocado roots infected with *P. cinnamomi*. The PR10 gene was identified in the response of Douglas -fir infected with *Phellinus weirii *[49]. The authors showed that very high concentrations of *Pin m III *(*PsemI *gene homologue) was responsible for resistance to the rust pathogen *Cronartium ribicola*. This *PR-10 *gene has also been used as a marker in screening for *P. weirii *resistance in Douglas-fir and could therefore be valuable in screening for PRR tolerance in avocado.

The gene encoding for the cytochrome P450-like TBP was the only transcript to be significantly induced by *P. cinnamomi *as early as 3 hpi. This enzyme features in oxidative metabolism and the production of ROS. This rapid response could be attributed to the universal nature of the protein in cell metabolism and growth. Additionally it has been reported to be involved in biotic and abiotic environmental responses as well as in the HR response to infection [44,50-52].

Our data revealed a noticeable host response at 12 hpi with the up-regulation of four transcripts, the *metallothionein-like *gene, the universal stress protein, *profilin *&*mlo*. Metallothioneins inhibit programmed cell death (PCD) and Fumonisin B1-induced root death in tomato infected with *Agrobacterium rhizogenes *through interference of the ROS pathway. ROS accumulation was significantly reduced under metallothionein over-expression, validating its function in ROS scavenging [53]. The significant induction of *metallothionein *in the highly tolerant avocado rootstock at 12 hpi implies that this protein may play a role in conferring disease tolerance to *P. cinnamomi *by scavenging ROS. ROS generation is indicative of the activity of the hypersensitive response (HR), which leads to cell death and is effective against biotrophic organisms [54].

The up-regulation of the universal stress protein at 12 hpi indicates the plant's response to the stress of infection by *P. cinnamomi*. Universal stress proteins are mediated by ethylene [55], and our results may therefore implicate the involvement of the ethylene pathway in response to *P. cinnamomi*, a pathway that has shown activation in the *Arabidopsis/P. cinnamomi *interaction [37].

Both *profilin *&*mlo *play a role in actin filament polarization [56,57] and actin rearrangement has been observed in plant-fungus interactions with successful pathogen infection resulting in the suppression of the rearrangement [58,59]. Profilin is known to localize to the site beneath the cell wall, that is penetrated by the oomycetous appresorium [60] and either promotes or prevents actin polymerization in the actin cytoskeleton [56]. Cell wall thickening during fungal attack also involves the re-orientation of actin filaments as a defense response in order to prevent pathogen ingress [61]. The up-regulation of *profilin *in avocado roots suggests that profilin is being produced in response to *P. cinnamomi *penetration. MLO, a transmembrane protein, modulates actin cytoskeleton polarization in resistant barley in response to a biotrophic fungus- *Blumeria graminis *f. sp. *hordei *[62]. Successful defence against pathogens results in cell wall strengthening and is correlated with increased actin accumulation at sites of attempted penetration [57].

The thaumatin-like gene expression showed no significant response to the oomycete infection. This might be explained by the fact that it is usually induced by viral, bacterial and fungal infection [46], and is believed to destroy fungal cell walls using a variety of enzymatic activities [63]. The LRR (leucine rich repeat) resistance protein-like gene also demonstrated no significant response to *P. cinnamomi*. Although resistance related LRR proteins have been found to interact specifically with other *Phytophthora *species, (*Solanum tuberosum-Phytophthora infestans *interaction) [64], no such interaction has to our knowledge been described for *P. cinnamomi *and its hosts, specifically avocado. However, the identification of a *LRR-like *gene from *P. cinnamomi *infected tolerant avocado roots warrants further investigation.

## Conclusions

This study identified an interesting set of genes regulated by the infection of the hemibiotroph *P. cinnamomi *by using 454 pyrosequencing. Defence genes included general defence-related transcripts (universal stress protein, *metallothionein *and *thaumatin*-like genes as well as cytochrome P450) and *Phytophthora *specific response genes such as (*thaumatin, PR-10 *and the *LRR resistance protein-like *gene) [44-47,49-52,55,64].

We inoculated avocado roots with a mycelial suspension, based on the genetic response by the plant, we hypothesize that the plant response is delayed due to the slower infection rate of mycelia as opposed to zoospores that are able to germinate and encyst within 2 hr of release from sporangia. Despite this delay five of the nine defence-related transcripts showed a significant early response to the pathogen between 3 and 12 hpi. Based on this first set of transcriptome data we hypothesize that the tolerance of the rootstock in this study is most likely polygenic and based on the early detection of *P. cinnamomi *followed by a response that included ROS and cell-wall strengthening. Ongoing research in our laboratory has generated a second set of transcriptome data and has included a variety of rootstocks with different levels of PRR tolerance and susceptibility as well as additional time-points.

We have successfully produced the first molecular data for the avocado-*Phytophthora cinnamomi *interaction and believe that this data will contribute to the understanding of host defence against this devastating pathogen thereby aiding in the selection of tolerant avocado rootstocks.

## Methods

### Plant material inoculation

Nine month old tolerant Dusa^® ^clonal avocado plantlets were provided by Westfalia Technological Services (Tzaneen, South Africa) and inoculated with a *Phytophthora cinnamomi *mycelial suspension containing mycelia and zoospores. A total of 33 g of mycelia was homogenised in 65 L of distilled water giving a final concentration of 0.5 g/L. This was then mixed into 112 kg of vermiculite in a mistbed. Plantlets were randomly grounded in vermiculite and constantly irrigated over a period of six weeks. Scanning electron microscopy and confocal microscopy were used to confirm *P. cinnamomi *infection, germination and root colonization which was then used to determine time points for root harvesting. Based on the results obtained, zoospores germinated within 1 hr & hyphae were visible at 6 hr, we selected our time points accordingly. Root material was harvested at 0 hour (uninfected), 3, 6, 12, 24, 48 and 72 hours post infection (hpi), snap frozen in liquid nitrogen and stored on dry ice (-78°C) until the root material could be transported back to a -80°C freezer. A subset of plants was left in the mistbed for six weeks as a positive control for disease.

### Generation of cDNA libraries for pyrosequencing

RNA isolations were done using the CTAB method [24]. Roots were ground in liquid nitrogen and 2-3 g of root material was used per RNA extraction. The Chloroform: isoamyl alcohol wash step was repeated 6 times followed by washing with ethanol thrice. Total RNA concentrations were quantified using the Nanodrop ND-100 Spectrophotometer (Nanodrop Technologies, Inc., Montchanin, USA) and verified on a 2% non-denaturing TAE agrose gel. Total RNA from three biological replicates (per time point) was combined before mRNA purification. Three technical replicates per biological replicate were performed for RNA isolation.

Prior to mRNA isolation, different time points were combined into three libraries for pyrosequencing and designated as uninfected, library 1 and library 2. The 0 hr time point and was regarded as the uninfected library with library 1 containing the 6 & 12 hr infection time points while library 2 contained the 24, 48 and 72 h time points. Purification of mRNA was done according to manufacturer's instructions using Oligotex (Oligotex™ mRNA kit, Qiagen, Valencia, California, USA). The mRNA purification was performed twice per sample to ensure the removal of any DNA contamination as well as to reduce the amount of rRNA available in the sample.

DNA contamination was assessed by using intron flanking primers. F3H forward:

(5'-TCTGATTTCGGAGATGACTCGC-3') and F3H reverse:

(5'-TGTAGACTTGGGCCACCTCTTT-3') to amplify a 300 bp fragment of the flavanone-3-hydroxylase gene from RNA as opposed to the 1200 bp fragment which is obtained from DNA.

cDNA libraries were synthesized with the Roche cDNA synthesis system (Roche Diagnostics, Mannheim, Germany) according to manufacturer's instructions and purified using the Qiagen MinElute PCR Purification Kit (Qiagen, Valencia, California, USA) before being sequenced. DNA contamination was assessed in cDNA as previously mentioned.

### Pyrosequencing and Bioinformatics

Libraries were sequenced by Inqaba Biotec (Sunnyside, South Africa) on the GS-FLX platform. Approximately 3 μg of cDNA was supplied for each library. A third of each library was tagged with a different ten nucleotide tag (Uninfected tag- 5'CGTGTCTCTA'3, library 1 infection tag- 5'CTCGCGTGTC'3, library 2 infection tag- 5'TAGTATCAGC'3) and sequenced on a single lane.

A total of 9950 reads were assembled into 371 contigs using the Newbler assembler version 1.1.02.15 (Roche). Reads were trimmed before contig assembly. Low quality reads were not included and the assembly was analyzed and annotated using dCAS (Desktop cDNA Annotation System) Version 1.4.1 Build 3791 and CLC Free Workbench software (CLC bio, Cambridge, MA). The BLASTX tool was used (using the PAM 30 matrix) in order to produce short and nearly exact matches. The sequence data generated in this study is available on the NCBI Transcriptome Shotgun Assembly Sequence Database BioProjectID: PRJNA72155. Gene sequences for genes quantified are available through accession numbers TSA JO840460-JO840468.

### Quantitative gene expression analysis

Nine genes were selected for gene expression analysis and included: *Thaumatin, thaumatin*-*like*, *metallothionein-like, leucine rich repeat resistance protein-like*, pathogenesis-related protein *PsemI*, *putative universal stress, profilin-like*, transmembrane protein *MLO *and *cytochrome P450-like TBP *(TATA box binding protein). Reference genes chosen for the study were *actin *and *18s *rRNA genes. The time points used to analyze the gene expression were 0 hours prior infection and, 3, 6, 12, 24 and 48 hours post infection.

cDNA synthesis was performed for qRT-PCR with starting material of 1-2 μg total RNA of each time point. The ImProm-II™ single strand cDNA system from Promega (Promega Corporation, Madison, Wisconsin, USA) was used in conjunction with random hexamer primers from Invitrogen (Invitrogen Life Technologies, Mississauga, ON, Canada). Reverse transcription was carried out under the following conditions: 25°C for 5 min, 42°C for 60 min and 70°C for 10 min.

### Quantitative PCR

Primers were designed using Primer Designer 4 for Windows, version 4.2^©^, (scientific and educational software, Durham, NC) based on sequence homology. Primers were designed to amplify products of no more than 150 base pairs, the selection was then auto adjusted using more aggressive criteria and primers were chosen within a 58-61°C range. Primers were synthesised by Southern Cross Biotechnology (Cape Town, South Africa) (Table 1).

Quantitative PCR was carried out using the Bio-Rad CFX96 real-time PCR detection system (Bio-Rad Laboratories, Hercules, CA). Reactions were performed in a 20 μl tube containing 5 μl of each cDNA sample, 10 μl of iQ SYBR Green supermix (Bio-Rad), 1 μl of each set of fragment specific forward and reverse primers and 3 μl SABAX water.

Thermocycling was carried out at 95°C for 10 min, followed by 55 cycles of 95°C for 10 sec an fragment specific annealing temperatures (Table 7) for 15 sec and elongation of 72°C for 15 sec. Three biological replicates were used with three technical replicates of each. The specificity of each primer pair was thoroughly investigated by standard PCR before the quantitative PCR was conducted and then verified by the presence of a single melting temperature peak. For the qPCR, the cycle threshold (CT) values were automatically determined using the accompanying Bio-Rad CFX manager (Bio-Rad CFX Manager™ version 1.5).

**Table 7:** Primer sequences of selected putative avocado defence-related genes from *Phytophthora cinnamomi *infected avocado roots.

Sequence ID	Fwd primer (5'-3')	Rev primer (5'-3')	Product length (bp)	Annealing °C
*Thaumatin *	CACCCTGTAGTTCACTCC	CCAGATGCTTACAGTTACC	75	58.5
*LRR resistance PLP *	GACATTCTTATAGCCATC	ATAAACAATCTGATTTTG	135	56
*Metallothionein like protein type 2*	AGTCTTCATCCCTAATACATATCCC	GTTTGTGCGTGTCTGGTTTC	76	58.5
*Thaumatin-like protein *	AAGCAGTCCTCAAGGTTC	TTTCCGTTAGTGTCAAAGC	79	65
*MLO protein *	TCGTGGATGGAAGGAGTG	ATGGGCAAATCTAAATCTTGTTG	85	58.5
*Pathogenesis-related protein PsemI *	GAAGATGGAGTACAAATAC	CACCTTGATGTGATAAAC	82	58.5
*Proflin-like protein *	TTCGGTATCTATGATGAG	ACGATATGACATTCAATAG	110	58.5
*Putative universal stress protein *	GACATTCTTATAGCCATC	ATAAACAATCTGATTTTG	135	56
*Cytochrome P450 like TBP *	GTCAAAGTGAAGAAATTC	AATCTCGTTAATCCATTC	119	58.5
*18S*	GTCAAAGTGAAGAAATTC	AATCTCGTTAATCCATTC		59
*Actin*	GAATCTGGACCATCTATTG	TACCAACCAAACCAAATC	114	58.5

Sequences were chosen from the library 1 and library 2 based on differential expressions. Two reference genes-actin and 18s were included.

### Statistical analysis

A Student T-test was carried out to determine significant differences between gene expression levels for quantitative gene expression analysis. Statistical analysis was performed using the JMP^® ^program version 9.0.0 (SAS Institute, Inc., Cary, NC) with a 95% confidence interval.

## Authors' contributions

WM and NVdB designed the experiments with the work being carried out by WM. Both authors prepared and approved the final manuscript.
